# How to Perform Cardiac Contrast-Enhanced Ultrasound (cCEUS): Part II—Advanced Applications and Interpretation

**DOI:** 10.3390/diagnostics15182371

**Published:** 2025-09-18

**Authors:** Harald Becher, Andreas Helfen, Guido Michels, Nicola Gaibazzi, Roxy Senior, Christoph Frank Dietrich

**Affiliations:** 1 Mazankowski Alberta Heart Institute, Edmonton, AB T6G 2B7, Canada; harald@ualberta.ca; 2 St. Marien Hospital, 44534 Lünen, Germany; a.helfen@t-online.de; 3 Notfallzentrum, Krankenhaus der Barmherzigen Brüder Trier, 54292 Trier, Germany; g.michels@bbtgruppe.de; 4 Department of Cardiology, University of Parma, 43121 Parma, Italy; ngaibazzi@gmail.com; 5 Royal Brompton Hospital, National Heart and Lung Institute, Imperial College, London SW7 2AZ, UK; r.senior@imperial.ac.uk; 6 Department General Internal Medicine (DAIM), Hospitals Hirslanden Bern Beau Site, Salem and Permanence, 3013 Bern, Switzerland

**Keywords:** echocardiography, contrast agent, ultrasound, ultrasound enhancing agent, cardiomyopathy, thrombus, tumor, myocardial perfusion

## Abstract

Ultrasound enhancing agents (UEAs, formerly called contrast agents) have enhanced echocardiographic diagnostics of myocardial disease and masses as well as myocardial perfusion abnormalities. This review provides up-to-date guidance on the procedures and interpretations according to current recommendations of imaging societies and considering the results of recent major studies. For the different indications, a standardized approach has been created including technical aspects, pre-assessment and primary scan planes, contrast-enhanced ultrasound (CEUS) procedure, interpretation and reporting. In a previous publication (part 1) the UEAs, imaging methods, preparation of the patients and assessment of global and regional LV function with UEAs were included. The two parts represent a comprehensive state-of-the-art compendium on how to perform CEUS examinations in clinical echocardiography and provide advice on education, qualification and quality control.

## 1. Introduction

Ultrasound enhancing agents (UEAs) have enhanced the assessment of myocardial disease and masses as well as the detection of myocardial perfusion abnormalities in clinical echocardiography [1,2,3]. The growing use of CEUS and recent studies have provided fresh insights on how to perform echocardiography with UEAs in clinical practice. In this article we translate the current knowledge into a comprehensive state-of-the-art compendium for the echocardiographer. We respond to the demands of clinicians for standardized and detailed advice which is specific to each indication. Echocardiographers also have a special need for detailed guidance on how to interpret and report studies with UEAs. However, there has been a lack of practical guidance, which we address in this article.

In a previous publication (part 1) [4] the UEAs, imaging methods, preparation of the patients and assessment of global and regional LV function with UEAs were covered.

The two parts represent a comprehensive state-of-the-art compendium on how to perform CEUS examinations in clinical echocardiography and provide advice on education, qualification and quality control.

### Echocardiography with UEAs for Assessment of Left Ventricular Aneurysm, Masses and Myocardial Disease

**Figure 1:**
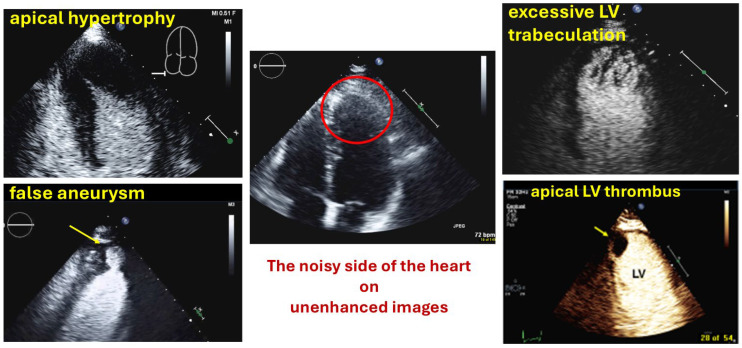
UEAs are particularly useful to enhance the display of pathology in the apical third of the LV where noise and artifacts are frequent.

The benefit of contrast echocardiography is most evident when assessing structures in the nearfield of the transducer. Nearfield noise or clutter artifacts are often present in non-contrast echocardiography and impair the display of pathology at the apical LV. When apical hypertrophy, aneurysms, non-compaction, thrombi or tumors cannot be ruled out or in, UEA should be administered (Figure 1, Table 1).

## 2. Left Ventricular Aneurysms

### 2.1. Background and Indications

True LV aneurysms are usually well displayed using non-contrast echocardiography [5]. However, detection of LV thrombi may be limited. False aneurysms usually occur acutely and are more difficult to delineate. Echocardiography with UEA should be considered when a false aneurysm is suspected, for better delineation of true aneurysms and accurate assessment of LV function or when thrombi need to be assessed in a true aneurysm [6].

### 2.2. Preparation and Performance

#### 2.2.1. Technical Aspects, Settings

Low MI contrast specific imaging methods are recommended, the same presets as for assessment of LV function (part 1) can be used [4]. Intermediate MI contrast specific imaging modalities should be used when no thrombus is displayed using the low MI contrast specific setting (for further details see the LV thrombus section).

#### 2.2.2. Pre-Assessment and Primary Scan Planes

**Figure 2:**
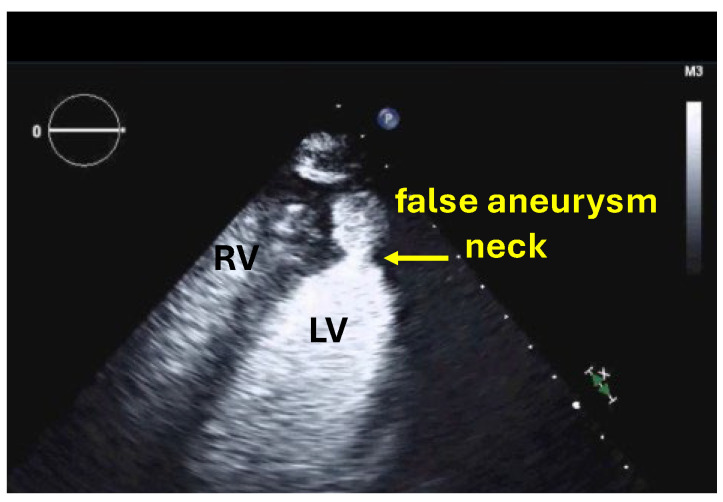
Apical false aneurysm—note the narrow neck compared to the diameter of the aneurysm, RV right ventricle, LV left ventricle.

**Figure 3:**
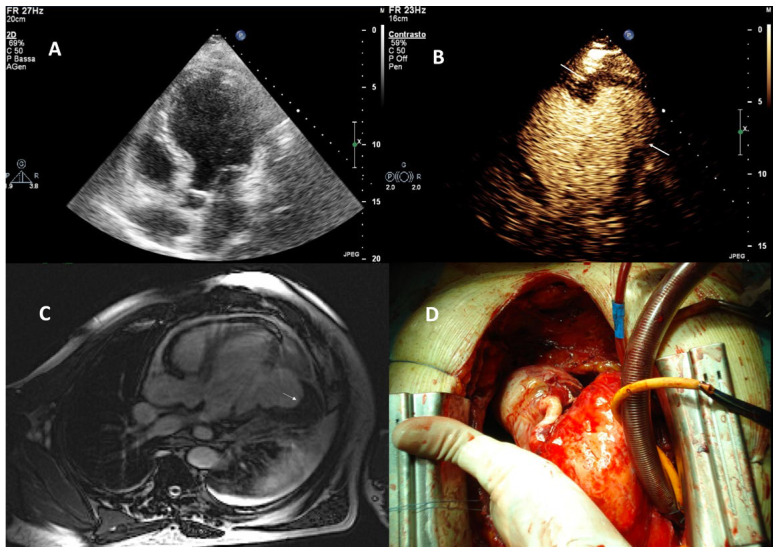
Large true aneurysm. Standard transthoracic echocardiography does not clearly visualize the post-MI lateral aneurysm (apical 4-chamber view) (**A**). After an intravenous bolus of UEA, CEUS reveals a massive lateral aneurysm (arrows) with suspect mural thrombus (apical 4-chamber view) (**B**). Cardiac magnetic resonance confirms the presence of the aneurysm and thrombus (arrow) (**C**). Intraoperative view of the aneurysm and thrombus (**D**).

Before injection of UEAs the LV should be scanned in the standard three apical views. The sector depth should be adjusted to include the entire LV plus a small section of the LA. Modified views should be recorded using zoom function and with the focus moved to the level of the aneurysm. In patients with apical aneurysms, a higher transmit frequency should be used to improve spatial resolution if the echocardiography machine has this option. When there is a suspicion of false aneurysm, color Doppler should be used to display blood flow in and out of the aneurysm through the usually narrow neck of the false aneurysm. Standard apical scan planes should be recorded, but for optimal display of the neck and full extension of the false aneurysm and possible thrombi in the false aneurysm, additional scan planes should be recorded by sweeping the probe (Figure 2 and Figure 3). Massive non-apical aneurysms, such as the post-MI true aneurysm shown in (Figure 3), may be missed by standard echocardiography. They can also be overlooked on UEA-enhanced echocardiography if off-axis views are not acquired. Off-axis views are facilitated by UEA enhancement of LV cavity signals.

#### 2.2.3. CEUS Procedure

The low-MI-contrast-specific method is recommended. The primary scan plane is the standard four-chamber view in which LV opacification is optimized (see Table 6, part I) [4]. After the standard four-chamber view, the examiner adjusts for the two-chamber view and the apical long-axis view. At least two loops with two or more cardiac cycles are recorded. In patients with apical aneurysms, further recordings should be performed in zoomed apical views using the intermediate MI method if a thrombus is not already displayed using the low-MI method. In addition to the long-axis recordings, short-axis sweeps through the apex can be helpful (Table 2).

### 2.3. Interpretation and Reporting

For false aneurysms the ratio of the neck diameter/aneurysm diameter—typically <50%—should be measured and reported (Figure 2). True aneurysms have a wide neck (Figure 3). If pericardial effusion is displayed in addition to the false aneurysm a thorough search for contrast signals in the pericardial effusion is necessary (see myocardial rupture). For both types of aneurysms, the size is determined by the maximum diameter. The global and regional LV function as well as the presence of thrombi are reported as outlined in the sections on LV function and LV thrombi.

### 2.4. Pitfalls

See assessment of left ventricular thrombi, Section 3.4.

## 3. Left Ventricular Thrombi and Tumors

### 3.1. Background and Indications

Most LV thrombi are attached to the LV apex and associated with apical STEMI, Takotsubo cardiomyopathy, dilated cardiomyopathy, aneurysms or endomyocardial fibrosis. Usually, LV thrombi are attached to severely hypokinetic/akinetic apical myocardium [7,8]. Thrombi attached to basal and mid-LV segments are less frequent but may be found attached to akinetic/dyskinetic wall segments. Thrombi along the mid/basal segments often can be displayed without UEAs. The focus should be moved to the depth of the akinetic/dyskinetic segments. The echogenicity of fresh thrombi can be very low, which limits the detection on recordings without UEAs. As thrombi have very few vascular components following UEA, they will appear as dark masses in the opacified LV. Tumors are vascularized and are opacified [9,10,11].

### 3.2. Preparation and Performance

#### 3.2.1. Technical Aspects, Settings for Echocardiography with UEAs

The same low-MI- contrast-specific imaging modalities as for assessment of LV function should be used. Intermediate-MI-contrast-specific imaging modalities should be used in addition when no definite diagnosis can be made with the default low-MI methods. The low-MI method should be used first, because it is the most sensitive method for detection of the UEA in the cavity. Often several scan planes can be recorded (loops with at least two cardiac cycles) with one bolus injection. The intermediate-MI-contrast preset can be useful (higher temporal and spatial resolution) in addition to the low-MI preset. The downside is more swirling due to microbubble destruction. Reducing the MI in the intermediate-MI-contrast preset reduces the sensitivity for detection of UEAs, which can be mitigated by injection of more UEA. If no thrombus is found in the standard apical views, sweeps should be performed to display the LV wall outside the standard views.

#### 3.2.2. Pre-Assessment and Primary Scan Planes

Before an injection of an UEA is considered for assessment of apical thrombus, the apical third of the LV should be scanned in the standard three apical views and modified views using zoom and with the focus moved to the apex. If possible, a higher transmit frequency should be used, which facilitates the detection/delineation of small trabeculations and thrombi. In the presence of akinetic apical segments, sweeps for assessment of mural thrombi should be performed. One should proceed with CEUS if LV thrombi cannot be ruled in or out with non-contrast 2D echocardiography.

#### 3.2.3. CEUS Procedure

**Figure 4:**
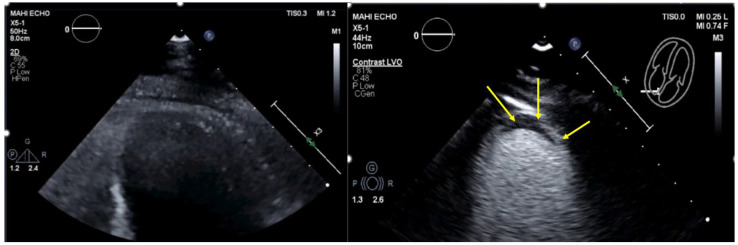
Thin apical crescent-shaped thrombus (arrows) attached to the apical myocardium (**right**). By slightly tilting the probe, the laminated thrombus could be distinguished from the myocardium. The thrombus was not detected on the corresponding non-contrast recording (**left**).

**Figure 5:**
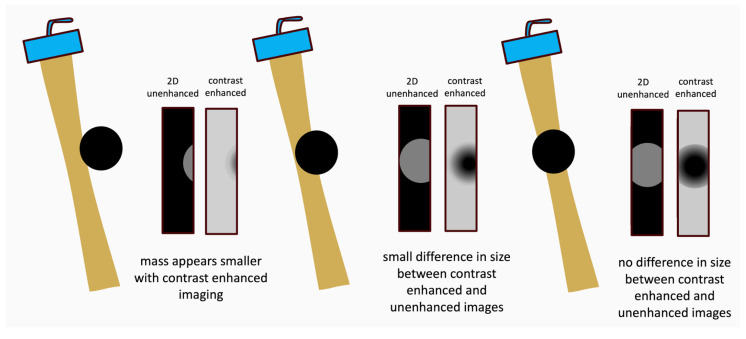
The elevation width of an echocardiographic transducer can be visualized by scanning through a contrast-enhanced liver. After a flash (which destroys the UEA in the elevation width) the transducer is rotated by 90 degrees, and the elevation width is displayed by a band without contrast. The elevation width is highlighted in orange color. The cartoon shows different positions of a thrombus (black) in relation to the elevation width (orange) of the ultrasound beam in 2D echocardiography. Note that the thrombus must be centered in the elevation width of the beam to avoid underestimation. The echocardiographer should gently tilt the transducer to search for the maximum extension of the thrombus.

**Figure 6:**
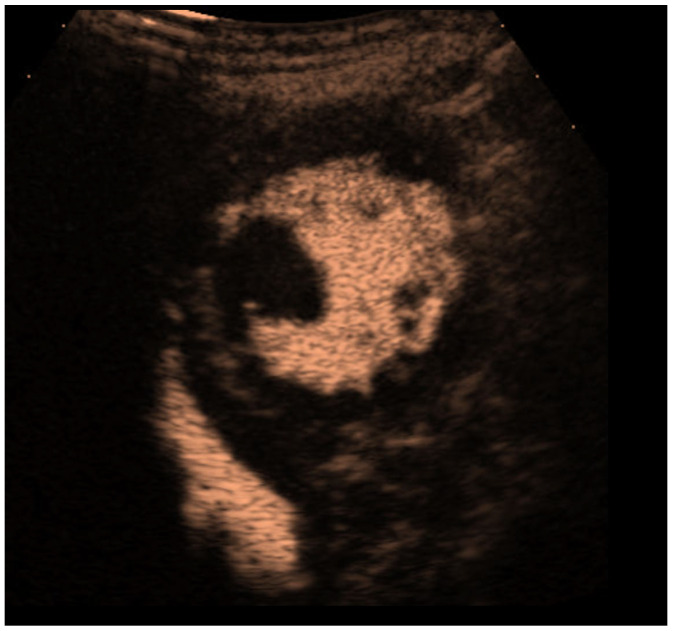
Left ventricular thrombus. Non-enhancing mass in the left ventricle (short-axis view).

**Figure 7:**
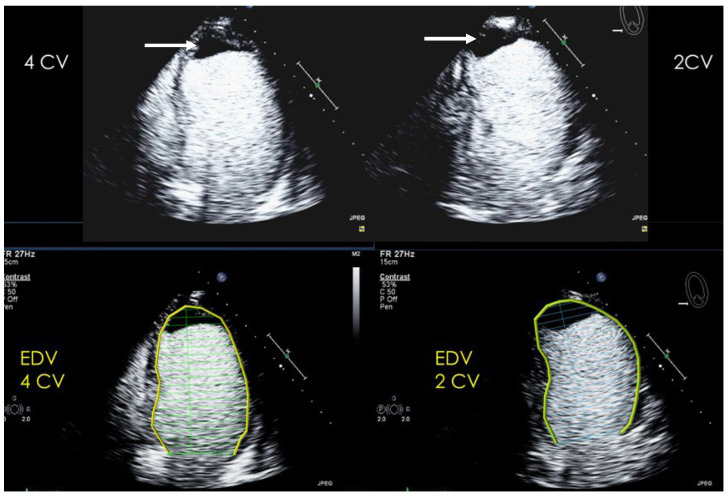
Measurement of LV volumes in the presence of a large apical thrombus (arrow). The thrombus must be included in the LV volume. This may require some interpolation as the border (green tracing) between thrombus and thrombus may not be well displayed at the attachment of the thrombus to the myocardium.

**Figure 8:**
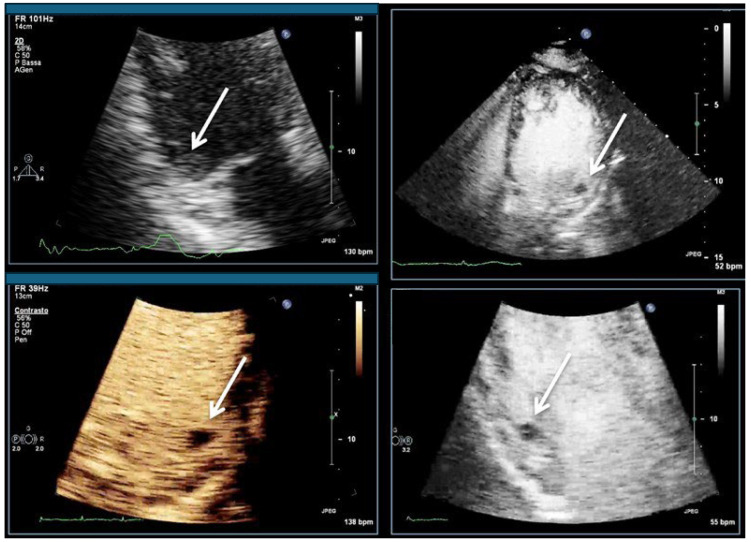
LV thrombus attached to the basal myocardium in a patient with infero-lateral myocardial infarct (arrows). Unenhanced 3-chamber view (**top left**), 4-chamber view (**top right**), modified 4-chamber view (**bottom left**), and 3-chamber view (**bottom right**).

**Figure 9:**
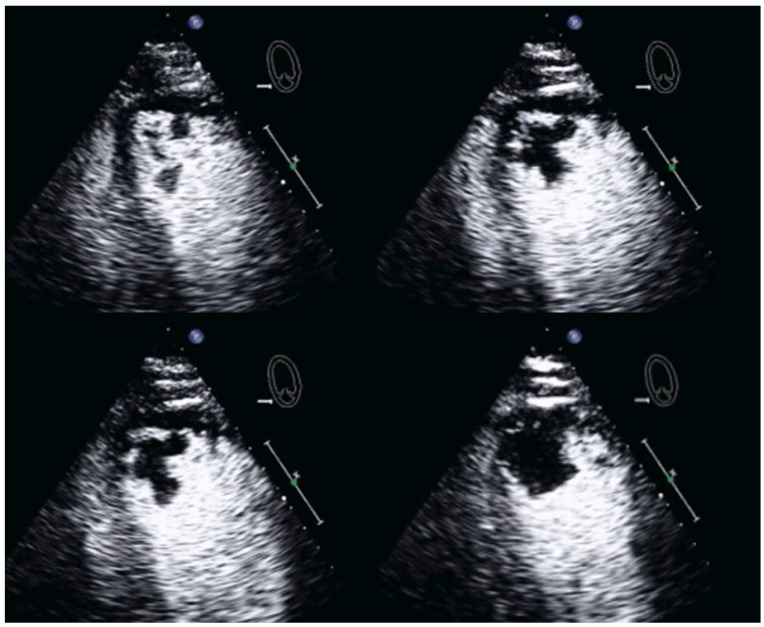
Apical sweep to evaluate the morphology of a thrombus: the frames show the irregular shape of the thrombus.

Recordings start using the low-MI method. The primary scan plane is the standard four-chamber view in which LV opacification is optimized according to Table 6 in part I [4]. Afterwards, the four-chamber view the two-chamber view and the apical long-axis view are imaged. At least two loops with two or more cardiac cycles are recorded. LV thrombi appear as unenhanced (dark) masses (Figure 4, Figure 5, Figure 6, Figure 7, Figure 8 and Figure 9). When no thrombi are visualized using the low-MI imaging modality, recording should be performed using the intermediate-MI modality. One should consider zoom of the apical LV or other regions with akinetic myocardium and placing the focus near the apex.

A thrombus seen in one imaging plane should be verified in another imaging plane or by biplane imaging. Recording of longer loops (>5 cardiac cycles) should be performed while sweeping through the apex. LV thrombi may be associated with excessive LV trabeculation. Sweeps and biplane imaging using intermediate-MI-contrast imaging help to distinguish thrombi from trabeculations (see pitfalls below). Thrombi often appear smaller on echocardiographic recordings with UEAs compared to non-contrast recordings. To display the maximum width, the thrombus must be centered in the elevation of the ultrasound beam (Figure 5).

### 3.3. Interpretation and Reporting

For diagnosis of LV thrombus, a mass should be attached to a hypo-/akinetic segment or aneurysm. The mass should be displayed in two orthogonal views and be without contrast signals. Cardiac tumors may be avascular. While opacification of a mass suggests a tumor, a lack of perfusion does not exclude tumors and does not establish thrombus as a diagnosis unless suspicion of thrombus is high (post-AMI, adjacent akinetic segments). In Table 3 the criteria are listed for reporting LV thrombi. For exclusion of LV thrombi optimal opacification of the recommended views is required. If this is not the case, it should be included in the report that suboptimal imaging limits the exclusion of LV thrombi and other imaging; in particular, MRI should be considered if this changes clinical management (Table 4). The presence of increased apical trabeculations should be reported because trabeculations can limit the detection of smaller thrombi.

### 3.4. Pitfalls

Apical swirling can limit the detection of apical thrombi. A reduction in the MI (0.02 steps) can be attempted but it may reduce the contrast signals. An additional bolus injection or increased infusion velocity is suggested. The downside is more attenuation/shadowing in basal parts of the LV. However, thrombi are usually searched for in the apical segments.

When LV contrast is too strong, thrombi may be ‘covered’ by signals from adjacent scan planes. Waiting long enough after bolus injection and establishing lower gain settings are required.

Thrombi are usually “echofree (black)”. Contrast signals within masses usually suggest vascularization, which is typical for tumors. Vascularization of chronic thrombi is rare. However, contrast signals within a thrombus may appear due to its irregular shape or the presence of crypts. These features can make adjacent blood appear as if it were inside the thrombus. Scanning in multiple planes with high spatial resolution (intermediate-MI-contrast-specific imaging) and adjustment of the gain help to display the thrombus without contrast signals.

When only part of a thrombus lies within the elevation width of the beam, its edges may appear brighter. This can lead to misclassification as perfused myocardium adjacent to the thrombus.

The size of the thrombus can be underestimated when the thrombus is not recorded in the center of the elevation width. Thorough review of all images recorded when sweeping through the thrombus is necessary to avoid underestimation of the size of the thrombus (Figure 5).

Muscular trabeculations may be taken for thrombi. Trabeculations are frequent in the apical LV cavity and can be prominent. When sweeping the imaging plane through the apex, the course of trabeculation can be followed; often, they connect with other trabeculations and are part of a network. Trabeculations are usually thin, but they may appear thick in certain views. Biplane imaging can help in these situations.

After acute myocardial infarction, it can take up to 11 days to develop LV thrombus. A follow-up echocardiogram after 10–14 days may be considered in patients with apical infarcts and akinetic segments.

### 3.5. Alternative Imaging Methods

When thrombi cannot be ruled in or out, cardiac MRI should be considered. Cardiac MRI has been shown to be more sensitive than echocardiography with UEAs. However, usually patients are not referred to MRI when no thrombus is displayed on a qualitatively adequate 2D echocardiogram with UEA. Cardiac CT is an alternative to MRI when MRI is not available.

## 4. Hypertrophic Cardiomyopathy

### 4.1. Background and Indications

The typical cavity pattern of apical hypertrophy may be visualized with non-contrast echocardiography. However, optimal image quality is crucial to detect apical aneurysm, which represents an increased risk of arrhythmias for the patients. Echocardiography with UEAs has been shown to provide a comparable sensitivity to MRI for apical aneurysms in hypertrophic cardiomyopathy [12] and is also indicated to rule in or out thrombi which can develop in the apical aneurysms. In addition, the measurement of LV wall thickness can be improved with UEAs [13]. Echocardiography with UEAs should be considered when apical hypertrophy is suspected or cannot be excluded. When there are clear signs of apical hypertrophy on 2D echocardiography (with and without aneurysm) a cardiac MRI should be arranged, looking for fibrosis and/or apical aneurysms not detected by echocardiography.

### 4.2. Preparation and Performance

#### 4.2.1. Technical Aspects, Settings

Low-MI-contrast-specific imaging methods are recommended, and the same presets as for assessment of LV function (part 1) can be used [4]. Intermediate-MI-contrast-specific imaging modalities should be used in addition when an apical aneurysm is visible and must be assessed for thrombus (for further details see the LV thrombus section).

#### 4.2.2. Pre-Assessment and Primary Scan Planes

Before an injection of an UEA is considered, the apical third should be scanned in the standard three apical views and modified views using zoom. The focus should be moved to the apex. If possible, a higher transmit frequency should be used. Color Doppler imaging can provide further suspicion of apical hypertrophic cardiomyopathy by showing high flow velocities in the residual apical cavity and myocardial vessels.

#### 4.2.3. CEUS Procedure

**Figure 10:**
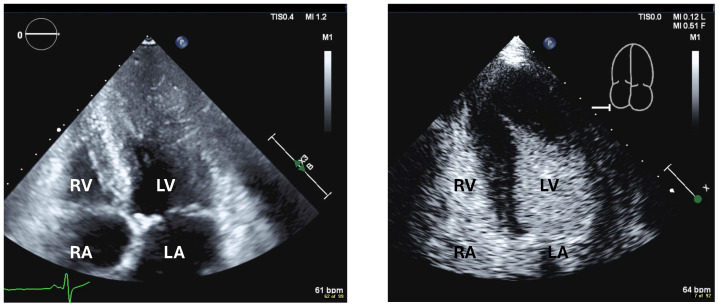
Apical hypertrophic cardiomyopathy, 4-chamber views, poor display of LV apex on unenhanced systolic fame (**left**), typical “ace of spades” apical contour on recording with UEA (**right**), thickened apical septum and apical lateral wall, no apical aneurysm displayed. RV right ventricle, RA right atrium, LV left ventricle, LA left atrium.

Recordings start using the low-MI method. Primary scan planes include the four-chamber view in which LV opacification is optimized according to Table 6 in part I (Figure 10). Then the two-chamber view and the apical long-axis view are imaged. At least two loops with two or more cardiac cycles are recorded. In patients with apical aneurysms, further recordings should be performed in zoomed apical views using the intermediate-MI method if a thrombus is not already displayed using the low-MI method.

### 4.3. Interpretation and Reporting

The typical apical shape of apical hypertrophic cardiomyopathy (hourglass or ace) is reported in addition to the size (maximum diameter) of the aneurysm and the presence and size of a thrombus inside the aneurysm (Table 5).

### 4.4. Pitfalls

Apical swirling should be avoided for optimal display of apical hypertrophy and detection of apical aneurysm.

Poor opacification of the LV cavity limits the detection of apical structural abnormalities.

In patients with apical aneurysm, see also pitfalls related to the assessment of thrombi.

## 5. Excessive Trabeculation of the Left Ventricle

### 5.1. Background and Indications

LV trabeculations are frequently displayed with non-contrast 2D echocardiography. In the past, LV non-compaction (LVNC) cardiomyopathy was suspected when there was an increased layer of the trabeculated myocardium in combination with a thinned compact myocardial layer [6,14]. According to a recent JACC: Cardiovascular Imaging Expert Panel Paper, the term “left ventricular non-compaction” is inaccurate and its use should be discouraged [15]. Incidental findings of excessive trabeculation with normal myocardial function and morphology in adults should not affect clinical management determined by other cardiovascular symptoms or abnormalities. In adults with hypertrophic or dilated cardiomyopathy, excessive trabeculation may be present but the extent of ventricular trabeculation has not been demonstrated to alter management or prognosis [15]. (Excessive LV trabeculations can impair the visualization of the compact myocardium and can limit the display of LV thrombi in patients with reduced LV function. As an expert opinion, echocardiography with UEAs should be considered when an accurate measurement of LV volumes and/or EF is required, or LV thrombi cannot be ruled in or out.

### 5.2. Preparation and Performance

#### 5.2.1. Technical Aspects, Settings

Low-MI-contrast-specific imaging methods are recommended to assess the global and regional LV function, and the same presets for assessment of LV function (part 1) can be used [4]. Using the low-MI preset may obscure the display of LV trabeculations. The myocardial trabeculations and additional LV thrombi are often better delineated using intermediate-MI-contrast-specific imaging modalities, which should always be applied in addition to the low-MI recordings.

#### 5.2.2. Pre-Assessment and Primary Scan Planes

Before an injection of a UEA is performed, the LV should be scanned in the standard three apical views and modified views using zoom with the focus moved to the apex. If available, a higher transmit frequency should be used. Color Doppler with reduced scale should be used to display accelerated blood flow between the muscular trabeculations in systole.

#### 5.2.3. CEUS Procedure

**Figure 11:**
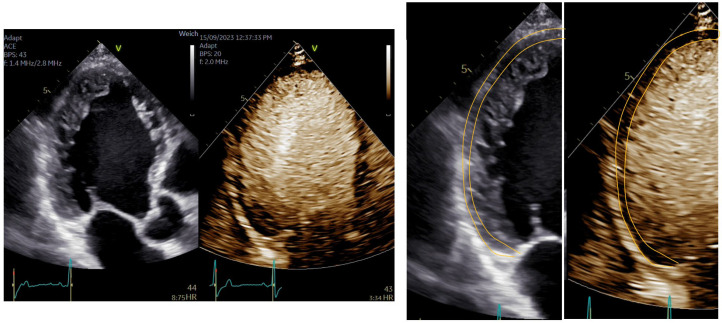
Apical 3-chamber view showing thin compact myocardium and excessive LV trabeculation; no thrombus was displayed. The yellow lines delineate the compact myocardial layer.

Recordings start using the low-MI method. The primary scan plane is the standard four-chamber view in which LV opacification is optimized according to Table 6 in part I. After the standard four-chamber view, the two-chamber view and the apical long-axis view are imaged (Figure 11). At least two loops with two or more cardiac cycles are recorded. Then recordings are performed using the intermediate-MI method according to the recommendations for LV thrombi.

### 5.3. Interpretation and Reporting

Measurement of the thickness of the trabeculated and compact myocardial layers has been performed in previous studies. However, according to the JACC: Cardiovascular Imaging Expert Panel Paper, quantification of the thickness of the compact and trabeculated myocardial layers does not change management [15]. In asymptomatic patients with normal diastolic and systolic function (EF and GLS), normal ECG and no family history of cardiomyopathy, more prominent and frequent trabeculation probably suggests a normal variant.

In patients with dilated or hypertrophic cardiomyopathy, recordings should focus on identifying LV thrombi. The severity of trabeculation itself is less relevant for management decisions (Table 6).

### 5.4. Pitfalls

See the above on “LV function” and “LV thrombi”.

## 6. Enhancement of Doppler Signals

### 6.1. Background and Indications

All UEAs enhance color and spectral Doppler signals [16,17]. When enhancement of poor Doppler signals is considered, the standard Doppler methods for non-contrast imaging are applied (Table 7).

Doppler methods are very sensitive for UEAs. After the injection of UEAs, the intensity of Doppler signals may become very high, causing saturation/blooming, which leads to overestimation of peak and mean velocities. Enhancement of valvular Doppler signals can be achieved using half of the dosages administered for LV opacification (see part I) [4]. When enhancement of Doppler signals is required in addition to LV opacification, the Doppler recordings should be performed after the LV recordings. No additional UEA injections are necessary. During the washout phase, the concentration of the microbubbles in the blood is still high enough to enhance the Doppler signals for several minutes.

### 6.2. How to Measure LAD Coronary Flow Velocity

The appropriate patient position: The patient should be placed in a true 90-degree left lateral position, more pronounced than the standard lateral position typically used for standard transthoracic echocardiography.

Probe positioning and the appropriate view: The standard 2D probe for transthoracic echocardiography can be used for practicality and to avoid probe switching. From a standard apical two-chamber view, move at least one intercostal space higher, with a slight tilt of the head of the probe towards the left shoulder; now the anterior sulcus is best viewed and the mid-distal LAD color Doppler signal is uncovered, using the appropriate color Doppler settings.

The appropriate settings: We recommend maximal wall motion filtering and optimization for low-velocity flow for the color and PW-Doppler presets, with color gain slightly higher than usual, and a low Doppler scale (around 20–30 cm/s). Color Doppler is first activated; under its guidance, the position of the mid-distal LAD is located, and then the PW-Doppler is activated to sample systolic and diastolic spectral Doppler velocity tracings (time–velocity integral often not perfectly depicted, but peak systolic and peak diastolic velocities are instead easily recognized in the tracings).

**Figure 12:**
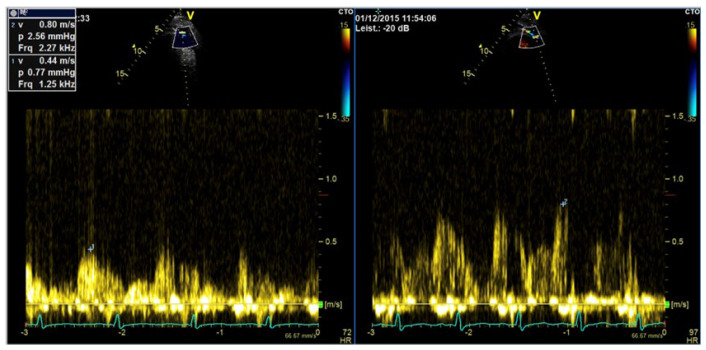
Measurement of coronary flow velocity reserve with contrast-enhanced PW Doppler; (**left**) diastolic flow velocity at rest; (**right**) diastolic flow velocity at maximum vasodilation with adenosine (170 micrograms/kg body weight). Flow velocity reserve 1.8, indicating stenosis of the LAD with or without microvascular dysfunction.

If the LAD cannot be located with standard color Doppler, a small bolus of contrast (e.g., 0.5 mL SonoVue^®®^) can be used, focusing on the anterior sulcus position. This makes LAD detection reliable, even for beginners, while maintaining standard Doppler settings. The mechanical index should be lowered to 0.1–0.2 (Figure 12).

This very low mechanical index makes 2D information almost disappear from the image (if not correcting with an increase in 2D gain), but contrast-enhanced LAD color flow is instead easily discernible. PW-Doppler is then activated using the same low mechanical index setting, as the final step for diastolic and systolic velocity measurement. While the use of contrast makes the LAD location process easy under color Doppler, it makes spectral Doppler velocity tracing rather noisy (scattering), so the quality of tracings is lower with contrast than without, but the quality remains sufficient for reliable peak velocity measurements.

### 6.3. Preparation and Performance for Enhancement of Valvular Flows

#### Technical Aspects, Settings

When UEAs are used for enhancement of valvular Doppler recordings, machine settings are not different from non-contrast Doppler recordings apart from lower gain settings (Table 8).

### 6.4. Velocity Measurements on Enhanced Doppler Recordings

**Figure 13:**
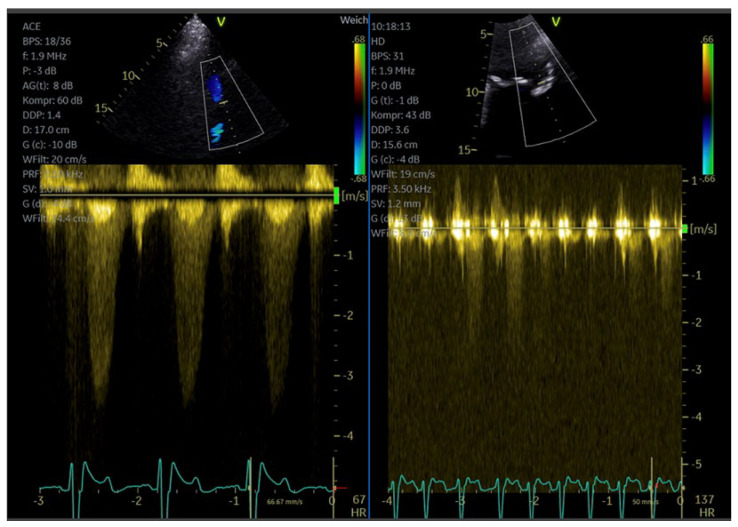
(**Left**) Contrast-enhanced CW Doppler recording of a patient with severe aortic valve stenosis, (**right**) unenhanced CW Doppler recording, Doppler envelops not closed, measurement of maximum velocity and velocity–time integral not valid.

The measurements and interpretation of peak and mean velocities are not different from Doppler measurements without UEAs. However, the gain often needs to be reduced to display the Doppler spectrum with normal brightness (Figure 13). No measurements should be made when the gain is too high (homogeneous brightness of the entire Doppler spectrum).

## 7. Myocardial Perfusion—Fundamentals and Practical Application

### 7.1. Background and Indications

Indications for assessing myocardial perfusion are listed in Table 8. For the assessment of myocardial perfusion and vascularity of tumors, the presence and kinetics of UEAs in the myocardial capillaries are reported [18]. The density of myocardial capillaries and the velocity of the blood flow through the myocardial vessels impact the contrast signals in the myocardium. At end-systole arterioles and venules are squeezed and blood is mainly found in capillaries, which comprise 90% of myocardial microcirculation.

In non-viable myocardial segments, most microvessels are lost. As a result, these regions fail to opacify after intravenous UEA injection. Stenoses of the epicardial coronary arteries and micro-vessel disease/dysfunction can reduce the velocity of the myocardial blood flow and capillary volume during stress [16]. However, myocardial blood flow is often still preserved at rest in patients with severe coronary stenoses due to the autoregulation (dilatation) of the resistance vessels. Abnormal perfusion can be assessed during physical and vasodilator stress: in segments with severe coronary stenoses, the increase in blood flow is limited because the microvascular resistance has already been reduced at rest and further reduction is limited. Therefore, contrast signals are reduced in ischemic segments during stress in comparison to segments with no obstruction of blood flow—best seen in the subendocardial layer (Table 9).

### 7.2. Preparation and Performance

#### 7.2.1. Technical Aspects, Settings

Myocardial perfusion is assessed using a *low-MI-contrast-specific imaging modality*. We suggest having a preset for myocardial perfusion imaging on the echo machine. The preset includes low-contrast-specific imaging, sector width/depth, MI for imaging, flash MI, number of flash frames and default gain. Most perfusion studies have used a flash MI of >0.7 for 5–10 frames. Also, the number of cardiac cycles recorded in a loop (at least 10) should be preset. Before starting the UEA infusion, one should check the background noise and look for residual signals of the myocardium and valves. Signals from fibrous tissue such as the mitral/tricuspid ring and pericardium can be seen, but myocardial signals should be minimal. The overall 2D gain should be maintained at around 65%. In patients with normal regional wall motion, the time gain compensation (TGC) may be adjusted so that myocardial and left ventricular opacifications appear uniform from apex to base [18]. This may require moving the nearfield TGC upwards.

After reaching the steady state during the UEA infusion and adjustment of gain, MI and/or infusion rate, the flash must be tested: An adequate flash results in a dark myocardium with the entire LV cavity still opacified (Table 10). The MI of the flash or the number of the flash frames is too high when there is incomplete LV opacification or swirling after the flash. The MI of the flash or the number of the flash frames is too low when contrast is still visible in the myocardium.

#### 7.2.2. Pre-Assessment and Primary Scan Plane

Before the start of the UEA infusion, the standard apical views should be displayed using 2D echocardiography and the focus placed at the mitral ring. When myocardial perfusion imaging is performed to assess the vascularity of a cardiac mass, additional scan planes may be necessary for optimal display of the mass.

#### 7.2.3. CEUS Procedure

Myocardial perfusion is assessed in apical four-, two- and three-chamber views. UEA infusion is recommended; the infusion rates for diluted Sonovue^®^/Lumason^®^, diluted Luminity^®^/Definity^®^ and Optison^®^ are listed in Table 3, part 1. About one minute after the start of the infusion, the concentration of microbubbles in the blood is stable and adjustments of the MI, gain or infusion rate can be made according to Table 10. The ‘best’ myocardial opacification is achieved when all normo-kinetic segments are opacified and endocardial borders are still visible. Due to the attenuation, the basal segments of the anterior wall and lateral wall may show less opacification compared to the other myocardial segments. Non-standard views can help enhance contrast signals in basal anterior and lateral segments. Positioning the lateral and anterior walls at the center of the sector is particularly useful. But the display of contrast signals may still be limited.

**Figure 14:**
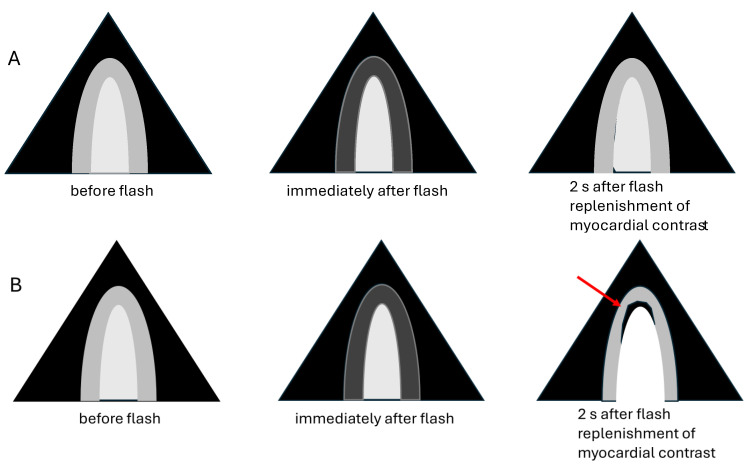
Flash–replenishment recordings for assessment of myocardial perfusion during stress. Normal myocardial perfusion: (**A**) immediately after the flash the myocardium becomes dark and within 2 s the contrast is replenished in the myocardium. Perfusion defect in the LAD territory (red arrow) (**B**). NOTE: perfusion defects may be visible without flash.

At least two flash–replenishment sequences are recorded in each of the apical views. A typical flash–replenishment recording includes 2–3 cardiac cycles before the flash and 10 cardiac cycles after the flash. During the flash the entire imaging sector becomes bright. Immediately after the flash the myocardium is dark because the microbubbles are destroyed in the imaging plane (Figure 14). During the following cardiac cycles, the microbubbles are replenished in the myocardial vessels. Finally, the intensity of the myocardial contrast reaches the same level as before the flash (Figure 14).

In stress echocardiography the same procedure is performed at maximum stress: For vasodilator stress with adenosine the UEA infusion is not interrupted. During dobutamine or exercise stress echocardiography, the UEA infusion often must be restarted at peak stress for recording of the flash–replenishment sequences. More detailed information on stress echocardiography protocols can be found in reference [19].

#### 7.2.4. Pitfalls

Inadequate myocardial contrast in all segments can be due to errors in contrast preparation (inadequate dosage, applying high negative or positive pressures) or use of incorrect machine settings.

**Figure 15:**
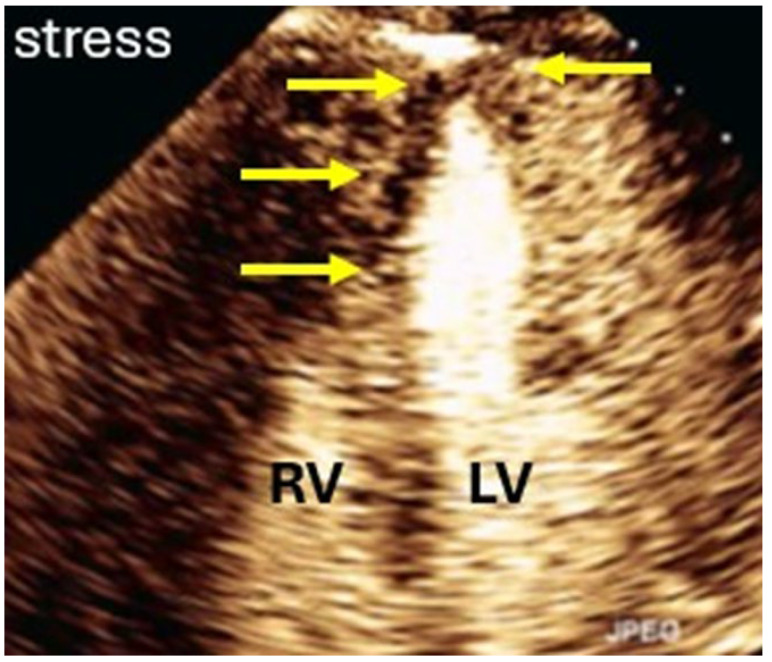
Four-chamber view with perfusion in the apex and apical septum defect (arrows) during dobutamine stress. Note the bright contrast signals in the mid septum and the lateral wall. RV right ventricle, LV left ventricle.

Inadequate myocardial opacification in apical segments can be improved by moving the focus towards the apex and/or by moving the nearfield TGC upwards [18]. True perfusion defects are usually located in the subendocardial myocardium (Figure 15). Pseudo-perfusion defects in the apical myocardium extend across the full wall thickness and are associated with contrast swirling in the apical LV cavity. Pseudo-perfusion defects are caused by increased destruction of the microbubbles in the nearfield and can be mitigated by reducing the MI in 0.02 steps.

Inadequate opacification of basal segments is due to the attenuation of the ultrasound. This mainly affects the basal segments of the anterior wall and lateral wall. Additional nonstandard views with the lateral and anterior wall in the center of the sector may be used to enhance the contrast signals.

Patients cannot hold their breath during the flash–replenishment sequence.

### 7.3. Interpretation and Reporting

A visual score can be used to assess myocardial opacification in each of the 17 myocardial segments at rest: 2: homogenous opacification within 5 s (viable myocardium); 1: heterogeneous opacification after 5 s (intermediate); and 0: no opacification (non-viable myocardium). Myocardial ischemia can only be evaluated during peak stress: when UEA replenishment takes longer than 2 s and a subendocardial or transmural is displayed.

At rest, akinetic LV segments with homogeneous myocardial contrast after myocardial infarction represent stunned myocardium. Akinetic myocardial segments with no opacification after UEA injection represent no reflow. In segments with no reflow, perfusion defects extend across the full myocardial thickness. In patients with acute STEMI, full wall thickness perfusion defects are also found before the infarct vessel is recanalized. Ischemic wall segments during stress or unstable angina have subendocardial perfusion defects.

The interpretation of myocardial perfusion must be performed in the context of the LV wall motion. Subendocardial perfusion defects are usually combined with wall motion abnormalities. A subendocardial perfusion defect during stress can facilitate the detection of a corresponding wall motion abnormality.

A perfusion defect at rest without corresponding wall motion abnormality is usually an artifact in patients who are asymptomatic at rest. Akinetic segments with fibrotic myocardium imply chronic infarction and display increased echogenicity. Fibrotic segments are often not suitable and in fact not required for assessment of myocardial perfusion. One may assess the viability based on wall thickness and wall motion during low-dose dobutamine stress.

Quantitative analysis can be performed by placing ROIs in the myocardium at rest and peak stress. Using the software of the manufacturers, the replenishment curves are obtained, and the myocardial blood flow can be quantified. A blood flow reserve (MBFstress/MBFrest) of >2 is normal.

However, this is a complex procedure. The ROI must cover the full myocardial thickness while excluding high-intensity endocardial and epicardial borders. In addition, corrections for cardiac translation are required [18]. The velocity reserve in the LAD is easier to perform (see enhancement of Doppler recordings).

**Figure 16:**
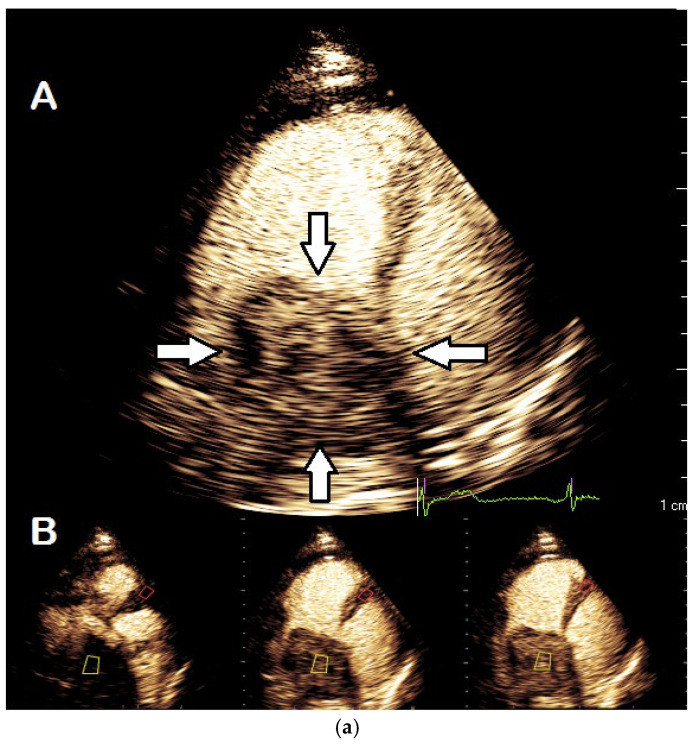

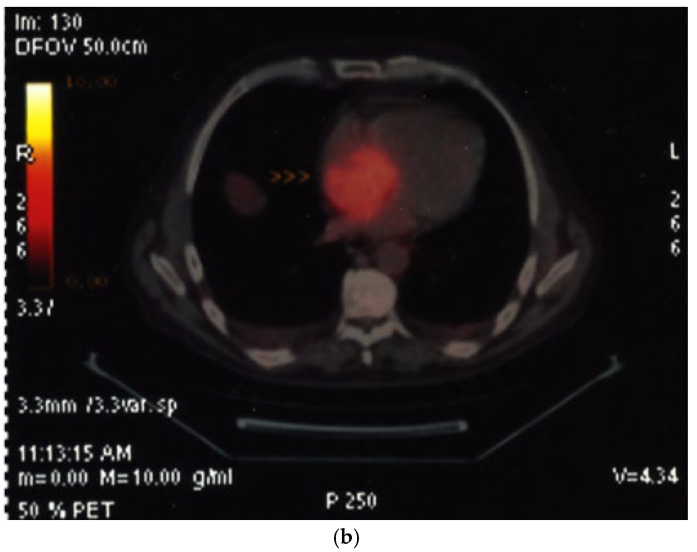
Right atrial mass (arrows) showing the heterogenous vascularization characteristic of malignancies, (**a**), zoomed still frame (A) and dynamic replenishment sequence (B, after flash) confirming significant vascularization of the mass, sample volume (yellow shape) in the centre of tumor for measurement of signal intensity). PET findings (**b**), histopathology and autoptic diagnosis was cholangio-cellular carcinoma.

For assessment of the vascularity of a cardiac tumor there is no standard visual classification. However, one should report the degree of opacification in comparison to the adjacent myocardium—less intense, similar or more intense (Figure 16). The disappearance of the contrast after flash and reappearance during replenishment prove the presence of vascularity and make thrombus unlikely (Table 11).

## 8. Echocardiography with UEA in Critically Ill Patients

There is good evidence on the benefit of using UEAs in critically ill patients where echocardiography without contrast agents is often suboptimal and other imaging methods are less feasible [20]. UEAs can be administered even in patients with end-stage renal disease. Echocardiography with UEAs has become a valuable tool for first-line imaging of patients with heart failure across the spectrum of patients with chronic heart failure to acutely ill patients [21]. The same protocols can be applied for critically ill patients as for patients with non-critical conditions. However, the echocardiography laboratories should be prepared for acute deterioration, and unstable patients should be monitored for 30 min after injection of the UEA.

**Figure 17:**
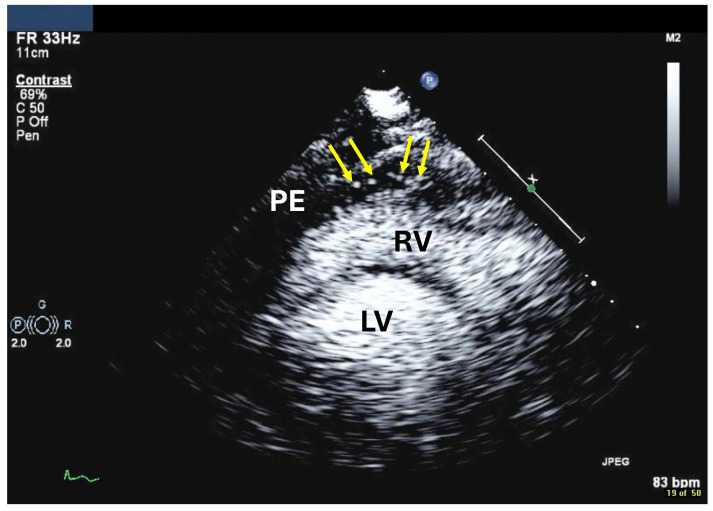
Contrast bubbles in the pericardial space (arrow) in a patient with bleeding into the pericardium, modified parasternal long-axis view, bubbles (arrows) are displayed anteriorly to the RV in the pericardial effusion (PE). NOTE: often only isolated contrast signals are displayed in the pericardial effusion. RV right ventricle, LV left ventricle.

Echocardiography with UEAs has been used to assess bleeding into the pericardial space, for example after coronary procedures, pacemaker insertion or myocardial rupture. The appearance of contrast in the pericardial effusion shows that the bleeding has not stopped (Figure 17).

One should always start with a subcostal four-chamber view to search for contrast signals in the pericardial fluid. The microbubbles float and tend to appear at the highest level in the pericardial space which is anterior to the RV (Table 12).

## 9. Education, Qualification

### 9.1. Qualification and Clinical Practitioner Training

Training in contrast echocardiography has become mandatory for sonographers and cardiologists. Physicians and sonographers should acquire knowledge on indications and contraindications, preparation and application of the different UEAs, imaging modalities, monitoring the patient during and after the exam, recognition and treatment of adverse events, and interpretation and reporting. Training should include immediate life support (ILS). Currently, there is no consensus on the minimum number of studies which must be performed and be reported. However, the authors suggest that echocardiographers (sonographers and physicians) perform and report at least 50 studies supervised by an experienced echocardiographer—usually the director of the echocardiography laboratory. To maintain competence, at least 20 echocardiography studies with UEA should be performed per year.

### 9.2. Quality Assurance and Control

Standard operating procedures (SOP) are recommended for the use of UEAs in the echocardiography laboratory as well as a logbook for collecting any documentation on adverse events. A recent multi-societal expert consensus statement on the safe administration of ultrasound contrast agents has provided an outline expert opinion on what constitutes appropriate supervision and the essential components of safe CEUS practice [22]. CME sessions of the echocardiography laboratory should include case presentations to discuss technical aspects and findings and to obtain opinions from experienced staff.

For quality assurance, we recommend periodic second reads and feedback. For example, five randomly selected cases every two years can be reviewed by an experienced echocardiographer to assess dosage, machine settings, and EF measurements.

## 10. Conclusions

This article elaborates the practical application of CEUS for specific echocardiographic questions when assessing patients for LV thrombi, tumors, myocardial disease and perfusion abnormalities. Systematic pathways have been created with detailed advice on every step of the CEUS procedures as well as on the interpretation and reporting of the findings. This article addresses the needs of echocardiographers who can integrate the pathways in their quality assurance. Standardized interpretation and reporting will improve communication between the echocardiographers with other medical staff. Finally, standardized procedures with adherence to high quality standards will facilitate the application of automated analysis tools and artificial intelligence for recordings with UEAs.

## Figures and Tables

**Table 1:** Indication for UEAs according to EACVI/ASE/BSE guidelines.

Myocardial disease and masses
Thrombi
Apical hypertrophy
Pseudoaneurysm
Myocardial rupture
LV diverticula
Excessive LV trabeculations

**Table 2:** Two-dimensional echocardiography with UEAs for assessment of aneurysms.

**Imaging method**	Low-MI-contrast-specific imaging and intermediate-MI-contrast-specific imaging
**Imaging planes**	Standard apical views, zoomed apical views and modified/zoomed views
**Sector depth/width**	First assessment of the global and regional function using low-MI method and display of the entire LV + 1/3 LA, then zoomed views to display the region of the aneurysm
**Focus**	At the depth of the aneurysm
**Gain**	Adjust to avoid obscuring the LV borders by intensive opacification of the cavity
**Contrast application**	Bolus injection
**Typical findings**	Clear demarcation from the normal myocardium Narrow neck of false aneurysms Search for associated thrombi and pericardial effusion (more likely near false aneurysms)
**Alternative imaging**	Cardiac MRI or cardiac CT in patients with contraindications to UEAs or inadequate recordings with UEAs

**Table 3:** Reporting LV thrombi/tumors.

Number
Localization: attached to which segments
Size: diameter in two planes, document in which planes measurements were performed
Shape: laminar, protruding, pedunculated
Mobility: yes/no
Comparison with previous echocardiogram if available

Vascularization of a tumor is assessed by comparing the opacification of the mass and the adjacent myocardium (less intense than, similar to, or more intense than the adjacent myocardium).

**Table 4:** Two-dimensional echocardiography with UEAs for assessment of LV thrombi. DD—differential diagnosis.

**Imaging method**	Low-MI-contrast-specific imaging and intermediate-MI-contrast-specific imaging
**Imaging planes**	standard apical views additional zoomed views sweeps through the region of the suspected thrombus
**Sector depth/width**	Entire LV + 1/3 LA to assess global/regional LV function, zoomed views of the region with the suspected thrombus
**Focus**	At the depth of the suspected thrombus
**Gain**	Adjust to avoid obscuring the thrombus by intensive opacification of the cavity
**Contrast application**	bolus injections
**Typical findings**	1. echofree mass 2. present in two different imaging planes 3. lump or laminar shape different from trabeculations’ documented size (two orthogonal diameters in at least one of the views), location, type (laminar vs. lump, single vs. multiple), surface (smooth vs. irregular) and mobility
**DD tumor vs. thrombus**	Opacified (vascularized) masses are suggestive of tumors; the vascularization can be demonstrated with myocardial perfusion imaging. CAVEAT: There are malignant tumors which appear avascular on CEUS. Tumors often are attached to normal myocardium.
**Alternative imaging**	*Thrombi:* Cardiac MRI when contrast echo is not possible or non-diagnostic, cardiac CT when cardiac MRI is not available *Tumors:* MRI provides tissue characterization and should be performed unless there is typical myxoma (attached to the interatrial septum)

**Table 5:** Two-dimensional echocardiography with UEAs for assessment of apical hypertrophy.

**Imaging method**	Low-MI-contrast-specific imaging, additional intermediate-MI imaging in case of apical aneurysm for assessment of thrombus in the aneurysm
**Imaging planes**	Standard apical views and apical sweeps to assess the aneurysm for thrombi
**Contrast application**	Bolus injections
**Typical finding**	1. Hourglass/ace-of-spades shape of apical LV cavity 2. Apical myocardium appears dark in late systole due to compression of the intramyocardial vessels 3. Apical aneurysms +/− thrombi in some patients
**Alternative imaging**	MRI useful for scar/fibrosis imaging and display of apical aneurysms CT in patients with contraindication for MRI and UEAs

**Table 6:** Two-dimensional echocardiography with UEAs for assessment of excessive LV trabeculation.

**Imaging method**	start with low-MI-contrast-specific imaging for assessment of global and regional LV function then use intermediate-MI-contrast-specific imaging which provides better display of LV trabeculations
**Imaging planes**	standard apical views parasternal and apical short-axis view
**Contrast application**	bolus injection
**Typical findings**	trabeculated layer apical, lateral, and inferior: more than 3 trabeculations, ratio of trabeculated/compact myocardium >2 The number of trabeculations and the ratio of trabeculated/compact myocardium does not seem to impact clinical management
**Alternative imaging**	MRI for tissue characterization in dilated cardiomyopathy and risk assessment in hypertrophic cardiomyopathy, assessment of LV thrombi when CEUS recordings are non-diagnostic

**Table 7:** Indications for enhancement of inadequate Doppler signals (incomplete/noisy Doppler spectrum unsuitable for tracing the envelope of the spectrum).

Flow assessment in epicardial coronary arteries (Color and PW Doppler)
Aortic stenosis (CW Doppler)
Pulmonary venous flow (PW Doppler) mitral regurgitation
Tricuspid regurgitation (CW Doppler) for measurement of PA pressure *

* If this is the only indication, agitated saline suffices.

**Table 8:** UEAs for enhancement of valvular Doppler recordings.

**Imaging method**	CW Doppler for aortic stenosis/tricuspid regurgitation PW-Doppler: for pulmonary–venous flow
**Imaging planes**	Same as for non-contrast Doppler imaging, the enhanced color Doppler signals facilitate the alignment of the cursor for spectral Doppler measurements
**Contrast application**	Bolus injection, the recordings can be performed during the washout after recordings for assessment of LV function
**Typical findings**	Intensive Doppler spectra Consider reduction in gain or power.
**Alternative imaging**	TEE, cardiac MRI

**Table 9:** Indications for assessment of myocardial perfusion with UEAs.

Viability in chronic CAD
No reflow in acute MI
Cardiac tumors
Assessment of myocardial ischemia/viability during stress echocardiography

**Table 10:** Adjustment of the mechanical index (MI) and the number of frames of the flash.

Adequate Flash	Flash Too Intense	Flash Too Low
Clears the contrast signals across the entire myocardium	Clears the contrast signals across the entire myocardium	Incomplete disappearance of myocardial contrast
Entire LV cavity still opacified	Major destruction of LV cavity contrast	Opacification of the entire LV cavity
	Reduce MI of the flash, e.g., 0.02 steps, or reduce the number of flash frames, e.g., 5 frames less	Increase MI of the flash, e.g., 0.02 steps, or reduce the number of flash frames, e.g., 5 frames more

**Table 11:** Two-dimensional echocardiography with UEAs for assessment of myocardial perfusion and vascularity of cardiac tumors.

**Imaging method**	Low-MI-contrast-specific imaging—Flash–Replenishment
**Imaging planes**	standard apical views additional modified views to optimize perfusion imaging in basal anterior/lateral segments or views to optimize the display of suspected tumors
**Contrast application**	Infusion bolus injection acceptable for demonstration of vascularization of a cardiac tumor
**Typical findings**	Normal myocardial perfusion: homogeneous opacification of the segment, contrast replenishment within 5 s after flash (2 s during stress) Abnormal perfusion: delayed contrast replenishment, perfusion defect, often combined with abnormal wall motion Tumors may show patchy opacification and include dark areas due to necrosis
**Alternative imaging**	Doppler velocity measurements (see above), nuclear imaging (SPECT, PET), cardiac MRI for assessment of myocardial perfusion and viability Tumors: MRI provides tissue characterization and should be performed unless there is typical myxoma (attached to the interatrial septum)

**Table 12:** Two-dimensional echocardiography with UEAs for assessment of bleeding into the pericardial effusion.

**Imaging method**	Low-MI-contrast-specific imaging, alternatively intermediate-MI-contrast-specific imaging
**Imaging planes**	First try subcostal view, then standard views
**Contrast application**	Bolus injection
**Typical findings**	Usually isolated bubbles in the pericardial fluid Review frame by frame!
**Alternative imaging**	Cardiac computed tomography with contrast

## Data Availability

The data presented in this study are available upon request from the corresponding author. The data is not publicly accessible, as the personal rights of the patients involved must be respected.
